# Chronic Kidney Disease: Decreasing Serum Klotho Levels Predict Adverse Renal and Vascular Outcomes

**DOI:** 10.1155/2024/2803739

**Published:** 2024-11-07

**Authors:** Abhijit Konnur, Sishir Gang, Umapati Hegde, Hardik Patel, Akash Pandya, Nitiraj Shete

**Affiliations:** ^1^Department of Nephrology, Muljibhai Patel Urological Hospital, Nadiad, India; ^2^Department of Biostatistics, Muljibhai Patel Urological Hospital, Nadiad, India

**Keywords:** chronic kidney disease, Klotho, vascular calcification

## Abstract

**Background and Objectives:** Soluble alpha Klotho (s.Klotho) is an emerging marker for chronic kidney disease (CKD) prognosis. The objective was to study the association between s.Klotho and CKD-related decrease in glomerular filtration rate (GFR), bone and vascular damage.

**Method:** A total of 118 patients with CKD stage 2–4 were enrolled and 107 patients continued in the study. Clinical and laboratory parameters were recorded at time of enrollment and 12 months. A double sandwich ELISA for s.Klotho was recorded in controls (*n* = 25) and patients' serum samples at 6 months (*n* = 107) and 12 months (*n* = 102). Primary endpoints like 40% or more fall in GFR, a requirement for renal replacement therapy (RRT), and death with different grades of s.Klotho deficiency were studied.

**Results:** Of the 107 patients (80 male and 27 female), mean s.Klotho was 3.46 ng/mL (02.3–04.2). The GFR fall was significantly different (*p* value < 0.0001) in the different grades of s.Klotho deficiency with Grade 4 s.Klotho deficiency (0.1–2.99 ng/mL) having the maximum fall of GFR at 9.2 mL/min/1.73 m^2^ (04.8–12.0) and minimum in Grade 2 (3–5.99 ng/mL) at 1.35 mL/min/1.73 m2 (03.0–02.75). The Ankle Brachial Pressure Index positively correlated with s.Klotho and the correlation coefficient was 0.536 (0.382–0.662) (*p* < 0.001). The carotid intimal medial thickness negatively correlated with s.Klotho and the correlation coefficient was −0.712 (95% CI: −0.797–−0.601, *p* < 0.001). All five deaths had s.Klotho Grade 4 (severe) deficiency. The event-free survival rate was maximum (100%) in Grade 2 Klotho deficiency and lowest (55%) in Grade 4 s.Klotho deficiency.

**Conclusions:** s.Klotho levels decreased significantly in patients with progressive kidney failure. s.Klotho levels significantly correlated with the presence of vascular disease. Death and need for RRT were significantly more in patients with severe s.Klotho deficiency.

**Summary**

• s.Klotho deficiency in chronic kidney disease is associated with bone and vascular disease and premature death.
• This study confirms that s.Klotho levels decrease significantly with progressive kidney failure and correlate with the presence of vascular disease.
• Death and need for RRT were significantly more in patients with severe s.Klotho deficiency.

## 1. Background

*α*-Klotho, popularly known as an antiaging gene, is mainly expressed in the kidney and plays a crucial role in human health [1]. Individuals with genetic s.Klotho deficiency exhibit phenotypic characteristics such as bone disease, vascular calcification, increased cardiovascular disease (CVD), elevated levels of fibroblast growth factor 23 (FGF23), hyperphosphatemia, and premature mortality that resemble the accelerated aging phenotype seen in patients with chronic kidney disease (CKD) [2–4]. Klotho deficiency in CKD may enhance renal tubule and vascular cell senescence, leading to endothelial dysfunction [5]. The dysregulated Klotho pathway is significantly associated with hyperphosphatemia, endothelial dysfunction, vascular cell senescence, and renal epithelial cell death, contributing to the development and progression of CKD [5–9]. A study was undertaken to define the role of s.Klotho in predicting CKD progression and specific complications, of mineral bone disorder, vascular dysfunction, and inflammation.

## 2. Methods

A single-center prospective observational study of patients with CKD stages 2–4 was undertaken according to the CKD EPI criteria with a study period of 18 months.

### 2.1. Inclusion Criteria

a. Age older than 18 years
b. Diagnosed with CKD by CKD EPI formula and CKD stages 2–4

### 2.2. Exclusion Criteria

a. Pregnant females
b. Patient on the transplant list
c. Patients with AKI in the last 3 months

### 2.3. Study Layout

A total of 118 eligible patients with CKD 2, 3, or 4 who consented to the study were enrolled between October 2021 and March 2022. Of these, 5 patients died during the study period and 102 patients completed the study period. The study flow diagram is given in the following.


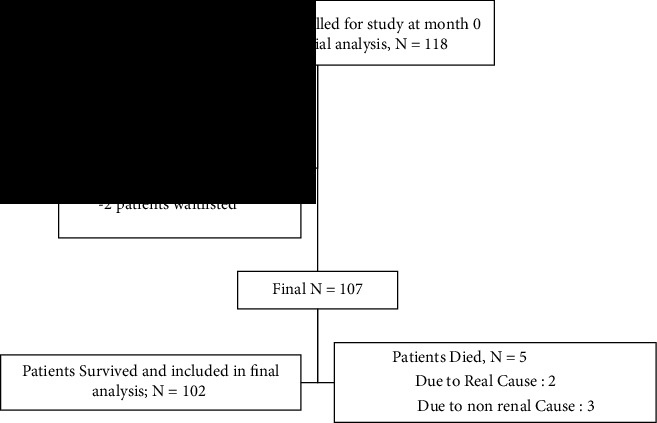


Demographic data such as name, age and sex of the patients were recorded upon enrollment. Clinical and laboratory parameters were recorded at enrollment and 12 months. The original plan was to record them at 0, 6, and 12 months, but due to the second wave of the COVID-19 pandemic, the second follow-ups of the enrolled patients were not possible, so all parameters were performed at enrollment and 12 months from enrollment.

Clinical parameters recorded were systolic blood pressure and the Ankle Brachial Pressure Index (ABPI).

Laboratory parameters included were (a) hemoglobin, serum albumin, CRP, ferritin, and % saturation of transferrin as markers of inflammation. (b) Serum creatinine and eGFR to look for the progression of CKD. (c) Serum calcium, phosphorus, and iPTH to look for metabolic bone disease. (d) Carotid intima media thickness (CIMT) to look for vascular health.

### 2.4. s.Alpha Klotho Methodology

At the time of enrollment and 12 months from the first visit, 4 mL serum samples were stored at −80°C for up to 9 months, and the tests were run first in June 2022 and then in March 2023. To determine s.Klotho levels in the non-CKD population, samples from 25 voluntary healthy prospective kidney donors aged between 18 years and 75 years with GFR > 90 mL and no proteinuria were collected. A double sandwich ELISA of stored serum samples of 92 patients in June 2022 by Human Alpha Klotho Kit by MyBioSource distributed by Biotechnolabs Delhi was performed.

The assay type was a Quantitative Sandwich with a detection range of −0.05 ng/mL–20 ng/mL and a sensitivity of 0.027 ng/mL. Intra-assay variability was CV < 8%, interassay CV < 10%, and normal assay value in humans > 12 ng/mL. The mean value in 25 control individuals was 12.8 ng/mL. Klotho levels of CKD patients were divided in 4 grades of deficiency to compare parameters and outcomes between different grades of Klotho deficiency (Table 1).

## 3. Methodology of CIMT and ABPI Measurement

A single blinded operator performed carotid Doppler ultrasonography. The transverse and longitudinal axes of the common carotid artery and carotid bifurcation were viewed from the neck of the patients at the supine position. CIMT was defined as the distance between the leading edge of the first and second bright lines. When an atherosclerotic plaque was present at the measurement site, it was included in the CIMT measurement. Plaques (defined as a protrusion into the lumen adding 50% to the thickness of the surrounding intima-media or maximal thickness of 1.5 mm in the carotid bifurcation or along the carotid arterial tree) were documented at 0 and 12 months. A normal median CIMT was defined as < 0.8 mm (0.61 in males and 0.59 in females) [10, 11]. ABPI was calculated using a validated blood pressure measuring device reading of highest systolic blood pressure in either of the brachial arteries and the posterior tibial arteries and calculating ratio of readings. A value of ≤ 0.9 was considered abnormal [12].

### 3.1. Statistical Analysis

This is a prospective observational study and the sample size computed was 106 patients, assuming CKD 2–4 prevalence rate of 8% and margin of error 8% at 5% level of significance. Collected information was stored in Excel and statistical analysis run with the help of IBM SPSS Version 25.0. All qualitative parameters are represented as frequency (percentage) while all quantitative parameters represented as the mean ± SD (min–max) and median (Q1–Q3). Further correlation analysis was done for Δ (s.Klotho) versus other quantitative parameters with the help of Karl Pearson's correlation coefficient and its significance was tested using the *t*-test. Scatter diagram has been used to show correlation graphically. Multiple linear regression analysis has been used to determine factors influencing fΔ (s.Klotho). ROC curve was used to know area under curve (AUC) and optimal cut point to determine the initial s.Klotho level, which predicts mortality.

## 4. Results

The average age was 51.04 ± 15.33 years with 78.4% males (Table 2).

### 4.1. Vascular Health

s.Klotho levels decrease with increase in ABPI (0.33 positive correlation with change in s.Klotho levels). Initial high s.Klotho levels showed decreased CIMT with 89% correlation. Changes in s.Klotho levels were significantly associated with increased CIMT with 53% correlation (*p* value = 0.005).

### 4.2. Inflammation

On correlation analysis, initial s.CRP significantly and negatively correlated with s.Klotho levels (−0.24 (−0.41–0.05)) (Table 1 Supporting Information). Also, change in CRP levels did not correlate with change in s.Klotho levels (Table 3). On multiple linear regression analysis, initial s.CRP levels were significantly and negatively associated with change in s.Klotho levels (Δ (S.Klotho)) (*p*=0.036). Serum albumin positively and significantly correlated with s.Klotho levels at initiation (*r* = 0.402 (0.20–0.527), *p* value: 0.0217) and at the end of the study (*r* = 0.442 (0.269–0.586), *p*=0.0001). Serum albumin levels were significantly higher in patients with higher s.Klotho compared with lower s.Klotho levels (*p* < 0.0001). Serum ferritin levels did not correlate with s.Klotho levels.

### 4.3. Kidney Health

Initial s.Klotho levels were significantly associated with subsequent GFR decline. A decrease in s.Klotho levels was significantly associated with a 32% decrease in GFR (*p* value = 0.002). Patients with diabetic nephropathy had a mean s.Klotho of 3.7 ng/mL and median fall in GFR of 6 mL/min/1.73 m^2^. Twenty five (23%) had undetermined etiology of CKD, a mean s.Klotho of 3.4 ng/mL, and median fall in GFR of 7 mL/min/1.73 m^2^. Sixteen (14%) had chronic glomerulonephritis, had a mean s.Klotho of 3.7 ng/mL, and GFR fall of 4.3 mL. Twelve patients with hypertensive nephropathy had a mean s.Klotho of 2.45 ng/mL and the median decrease in GFR was 7 mL. Six patients had other causes of CKD, 5 had obstructive nephropathy, and 1 had ADPKD. The mean s.Klotho was 3.9 ng/mL and median GFR fall were 5.2 mL.

There was a significant strong positive correlation observed between Δ s.Klotho and Δ eGFR (*r* = 0.75; *p* value < 0.001). At initiation, 10 (9.4%), 41 (38.3%), and 56 (52.3%) patients were in CKD stages 2, 3, and 4, respectively. At the end of the study, 5 patient died and out of surviving, 102 patients, i.e., 5 (4.9%), 35 (34.3%), 41 (40.2%), and 21 (20.6%) were in CKD stages 2, 3, 4, and 5, respectively.

**Figure 1:**
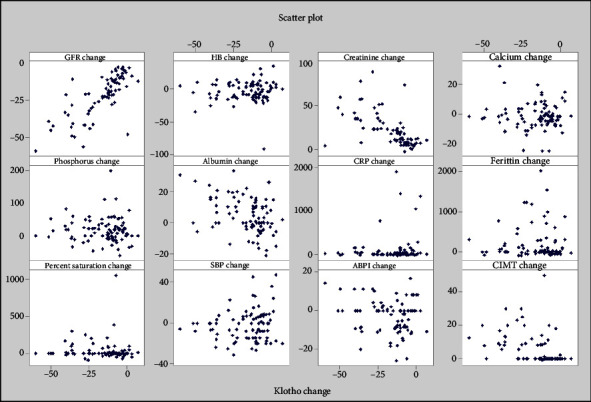
Scatter plot of Δ (s.Klotho) vs. other parameters.

A further significant negative but weak correlation was observed between Δ s.Klotho vs. Δ s.albumin (*r* = −0.428; *p* < 0.001) and Δ (CIMT) (*r* = −0.402; *p* < 0.001). Age-related Klotho level change Δ (s.Klotho) was not significant (Figure 1).

**Figure 2:**
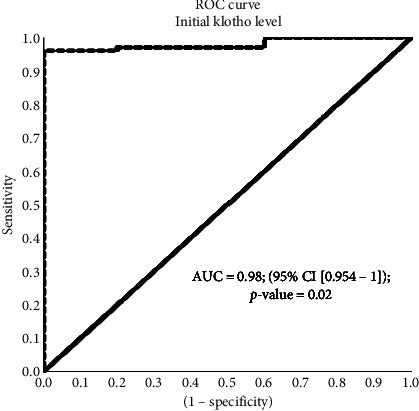
ROC curve for serum initial Klotho level.

The AUC for initial s.Klotho level is 0.98 (95% CI [0.954–1]; *p* value = 0.02), which is significant. Also, from the coordinates of the ROC curve for the initial s.Klotho level, cut point s, Klotho level < 1.7 showed significant mortality with Sn and Sp as 94% and 100%, respectively (Figure 2).

On multiple linear regression using baseline factors which influence ser.Klotho levels (Δ (s.Klotho)), ser.CRP, iPTH, ABPI, and CIMT emerged as significant factors which are associated with change in ser.Klotho levels (Table 4).

A fitted linear regression model (Table 5) to determine influence of different grades of s.Klotho deficiency on 1-year outcomes has predicted that patients with Grade 1 Klotho deficiency have < 2% GFR fall, Grade 2 Klotho deficiency have 2%–13% fall in GFR, Grade 3 Klotho deficiency have 13%–24% fall in GFR, and Grade 4 Klotho deficiency have > 24% predicted fall in GFR. Similar results were seen for CIMT and s.albumin.

## 5. Discussion

This study was adequately powered, with 107 patients (80 males and 27 females) participating in the study.

### 5.1. s.Klotho and Progression of CKD

eGFR levels significantly and directly correlated with s.Klotho levels at the initiation and end of the study (*p* value = 0.0026 and *p* value = 0.0012, respectively). The GFR fall was significantly different (*p* value < 0.0001) in the different grades of s.Klotho, being the highest in Grade 4 of s.Klotho deficiency (0.1–2.99 ng/mL) with 9.2 mL/min/1.73 m^2^ (04.8–12.0) and lowest in Grade 2 (3–5.99 ng/mL) with 1.35 mL/min/1.73 m2 (03.0–02.75). The % GFR fall strongly and positively correlated with the s.Klotho percent fall (*p* < 0.0001), suggesting a more severe fall in s.Klotho with a severe fall in GFR. Patients with different tertiles of s.Klotho levels had significant differences in fall in GFR (between low, average, and high s.Klotho levels (*p* < 0.0001, CI 95%)). Lower serum *α*-Klotho levels were associated with a more severe CKD stage in a cross-sectional analysis [3].

• s.Klotho and primary outcomes: death, 40% reduction in GFR, or initiation of RRT.
  It was observed that over 12 months of study, event-free survival was higher (100%) in Klotho Grade 2 deficiency (9-6 ng/mL) patients (*N* = 4). The event-free survival was 91% in Klotho Grade 3 (3–5.99 ng/mL), where 5 out of 56 patients reached ESRD. In Klotho Grade 4 deficiency (< 3 ng/mL), event-free survival at 12 months was 55%. Eighteen out of 47 patients reached ESRD and five out of 47 patients died. Two out of 5 deaths were renal death.
  In a study conducted by Eleni Manou et al. during a median follow-up of 36 months, 40 (31.2%) participants reached the primary endpoint (31 initiated renal replacement therapy and 9 died) [13].
• s.Klotho and inflammation.
  In this study, s.CRP, serum albumin, and serum ferritin levels were considered as markers of inflammation.

### 5.2. s.Klotho and MBD

In this study, there was no correlation among serum calcium, serum phosphorus, and calcium phosphorus product ratio. We also did not find any correlation between s.PTH and s.Klotho levels.

In a previous study by Pavik et al., a positive correlation between s.Klotho, calcium, and Vitamin D3 levels and a negative correlation between s.Klotho and FGF23 levels and serum phosphorus levels were seen (Table 6). But after adjusting for GFR and age, that correlation became attenuated [14]. In another study by Rotondi et al., s.Klotho correlated negatively with s.PTH (*ρ* = −0.28, *p* < 0.05) and positively with Ca (*ρ* = 0.30, *p* < 0.01) while no correlation was found between s.Klotho and 1,25OH D3. FGF23 levels correlated positively with PTH (*ρ* = 0.43, *p* < 0.001) and Ps (*ρ* = 0.51, *p* < 0.001) [18].

Therefore, the actual relationship between s.Klotho and MBD may be more complex than previously thought.

### 5.3. s.Klotho and Vascular Health

In this study, ABPI and CIMT were significantly associated with s.Klotho levels, both at the initiation and end of the study (*p* < 0.0001). In an analysis by tertiles, ABPI was significantly lower in the low s.Klotho tertile (*p*=0.001) and CIMT was significantly higher in the high s.Klotho tertile (*p*=0.001).

Initial mean ABPI was 1.09 ± 0.17 (0.1–1.4), which reflected the absence of peripheral vascular disease in the patients. ABPI levels positively correlate with s.Klotho levels. The mean ABPI was higher in s.Klotho Grade 2 than in Grades 3 and 4, and the difference was maintained throughout the study period (*p* < 0.0001). This implies increased vascular disease in patients with lower s.Klotho levels.

In a cross-sectional study conducted by Correa et al. on 106 patients with CKD 3-4, patients with subclinical atherosclerosis presented with lower serum levels of Klotho. Both variables were associated with the presence of subclinical atherosclerosis, being directly related to ABPI and inversely related to CIMT (*p* < 0.0001 for both). Multiple regression analysis demonstrated that both variables were significant determinants of ABPI (adjusted *R*2 = 0.511, *p* < 0.0001) and CIMT (adjusted *R*2 = 0.445, *p* < 0.0001) independent of traditional and emergent cardiovascular risk factors. Moreover, both constituted protective factors against subclinical atherosclerosis (OR: 0.993 (*p*=0.002) and 0.231, respectively [*p*=0.025]) [19].

Initial median CIMT levels of 0.9 are a reflection of poor vascular health. CIMT levels at the beginning and end of the study were significantly higher in s.Klotho Grade 4 patients than in Grade 2 and Grade 3 patients (*p* < 0.0001). CIMT was higher in patients with Klotho Grade 4 at initiation and at the end of the study, there was also an increase of 0.1 mm in mean CIMT in Grade 4 Klotho at 1 year, showing progression of disease.

CIMT is an operator dependent but fairly strong quantitative marker of atherosclerosis, and ABPI is an inexpensive, yet reproducible parameter of vascular calcification. In our observations, s.Klotho strongly predicted atherosclerosis (especially in Grade 4). It would be interesting to see the association of changes in these atherosclerosis markers in either direction with changes in s.Klotho levels as an effect of treatments given either for CV risk reduction or for slowing CKD progression, which we could not observe in the short time frame of our study.

### 5.4. Limitations

Being a single tertiary care center study, there is a nonuniform representation of CKD 3 and 4 patients with a lower number of patients in the CKD 2 and Klotho Grade 2 groups, leading to skewed data toward low GFR and median tertile toward lower levels.

The 1-year duration of the study may be short to observe measurable changes in chronic events, such as worsening vascular disease.

Serum levels of Klotho are not defined for the Southeast Asian population for healthy or CKD populations; therefore, population-based studies are needed with representation from different heterogeneous populations to define normal and abnormal levels of Klotho.

We did not measure FGF23 levels with s.Klotho levels, which are closely related to s.Klotho and better help in associating Klotho with MBD status. We did not have enough patients with different grades of Klotho in different severities of proteinuria to comment on the association of s.Klotho and fall in GFR, considering proteinuria is an established risk factor for worsening renal function.

## 6. Conclusion

Decreasing soluble alpha Klotho levels in CKD patients significantly correlated with progression of CKD, vascular disease, and mortality but not with metabolic bone disease. Larger studies with different ethnicities are required to determine whether modulating s.Klotho levels may influence the outcomes in CKD.

## Figures and Tables

**Table 1:** Grading of s.Klotho deficiency based on serum Klotho levels.

Grade of deficiency	Serum Klotho levels (ng/mL)
Grade 1	> 9
Grade 2	9–6
Grade 3	3–5.99
Grade 4	< 3

**Table 2:** Initial and final descriptive measurements of study parameters.

Parameter	Initial measurements (*N* = 107)	Final measurements (*N* = 102)
Sex: male: female (%)	80: 27 (74.8: 25.2)	—
Age (in years)	51.04 ± 15.33 (14–83) **53 (39–63)**	—
DM	49 (45.79%)	49 (48.04%)
HTN	68 (63.55%)	57 (55.88%)
GFR	33.32 ± 18.63 (10–116) **28.5 (18.25**–**45.5)**	28.09 ± 16.96 (5–116) **26 (15.78-37.75)**
A.Klotho	3.63 ± 1.83 (0.29–12) **3.43 (2.49**–**4.38)**	3.32 ± 1.78 (0.5–10) **3.20 (2–4.2)**
HB	11.51 ± 1.64 (8.6–16.2) **11.5 (10.5**–**12.4)**	10.9 ± 1.52 (1–14) **11 (10-12)**
Creatinine	2.57 ± 1.21 (0.46–6.92) **2.38 (1.76**–**3.27)**	2.88 ± 1.25 (0.6–6.9) **2.75 (2-3.8)**
Calcium	9.46 ± 0.74 (7.33–13.14) **9.53 (9**–**9.86)**	9.37 ± 0.68 (7.33–13.14) **9.46 (9-9.74)**
Phosphorus	4.06 ± 1.23 (1.81–10) **3.72 (3.23**–**4.66)**	4.68 ± 1.32 (1.32–10.15) **4.58 (3.97-5.2)**
Product ratio Ca∗Po	38.49 ± 12.11 (16.89–100) **35.8 (30.64**–**42.5)**	—
Albumin	3.88 ± 0.54 (2.23–4.63) **4 (3.56**–**4.33)**	3.76 ± 0.47 (2.57–4.86) **3.89 (3.5-4.02)**
Cholesterol	174.88 ± 58.74 (18.63–288.11) **178.42 (141.34**–**209.63)**	—
LDL	103.6 ± 44.71 (36.8–205.48) **92.19 (70.27**–**133.33)**	—
HDL	38.96 ± 10.63 (22–72) **38 (31**–**46.5)**	—
Triglyceride	190.44 ± 102.5 (48–527) **176.41 (126.58**–**216.04)**	—
FBS	111.24 ± 43.62 (85.36–272.26) **98.84 (89.4**–**119.53)**	—
HbA1c	7.16 ± 1.4 (5–10.3) **7 (5.85**–**8.1)**	—
CRP	15.13 ± 31.32 (0.1–266.3) **5.8 (1.8**–**14.6)**	16.79 ± 34.13 (0.5–300) **7 (3.25-20)**
Urine protein	133.35 ± 181.05 (1.9–872) **76.55 (18.75**–**182.88)**	—
PTH	184.07 ± 181.68 (7.14–1131.3) **125.6 (78.5**–**230.1)**	—
Ferritin	101.26 ± 109.9 (3.2–535.1) **69.75 (29.88**–**128.15)**	153.52 ± 134.05 (4–800) **120 (60-200)**
% Saturation	21.36 ± 15.35 (2.16–76) **18.8 (11.4**–**24.45)**	23.91 ± 11.2 (3–60) **25 (15-29.5)**
SBP	142.38 ± 19.01 (110–190) **140 (130**–**150)**	137.22 ± 16.68 (110–180) **140 (120-150)**
ABPI	1.09 ± 0.17 (0.1–1.4) **1.1 (1**–**1.2)**	1.07 ± 0.15 (0.7–1.4) **1.1 (1-1.2)**
CIMT	0.89 ± 0.25 (0.4–1.8) **0.9 (0.7**–**1.1)**	0.94 ± 0.3 (0.4–1.9) **0.9 (0.7-1.2)**
Uric acid	7.22 ± 2.26 (2.31–15.89) **7.05 (5.96**–**8.42)**	—
CKD stage (%)		
II	10 (9.34)	5 (4.90)
III	41 (38.32)	35 (34.31)
IV	56 (52.34)	41 (40.20)
V	—	21 (20.59)
Death	—	5 (4.67)

*Note:* Note that all quantitative parameters are represented by the mean ± SD (min–max) and median (Q1–Q3). The median values are printed in bold in both the tables.

**Table 3:** Δ (s.Klotho) correlation with other parameters.

Δ (s.Klotho) versus	Correlation (95% CI)
Δ (eGFR)	0.75∗ [0.62–0.89]
Δ (HB)	0.08 [−0.12–0.27]
Δ (s.Creatinine)	−0.71∗ [−0.85–−0.57]
Δ (s.Calcium)	0.06 [−0.13–0.26]
Δ (s.Phosphorus)	−0.03 [−0.23–0.17]
Δ (s.Albumin)	−0.43∗ [−0.61–−0.25]
Δ (s.CRP)	0.14 [−0.05–0.34]
Δ (s.Ferritin)	0.02 [−0.2–0.23]
Δ (% Saturation)	−0.11 [−0.37–0.16]
Δ (SBP)	0.14 [−0.06–0.33]
Δ (ABPI)	−0.09 [−0.29–0.1]
Δ (CIMT)	−0.41∗ [−0.58–−0.23]

^∗^Statistically significant correlation at a 5% level of significance.

#Δ: difference between initial and final measurement, i.e., (initial–final).

**Table 4:** Multiple linear regression for predicting factors for Δ (s.Klotho).

Model parameters	Coefficients		*p* value
	*B*	Std. error	
(Constant)	−42.115	81.987	0.609
GFR initial	0.094	0.105	0.372
Hb	−0.130	0.936	0.890
Ser. calcium	5.125	9.176	0.578
Ser. phosphorus	13.472	19.302	0.488
Calcium phosphorus product ratio	−1.679	2.073	0.421
Ser. albumin	0.059	3.083	0.985
Ser. C-reactive protein levels	−0.081	0.038	**0.036**
Ser. iPTH levels	0.020	0.009	**0.030**
Systolic BP	−0.104	0.079	0.197
ABPI	16.380	7.448	**0.032**
CIMT	−27.766	5.878	**< 0.001**

*Note:* Baseline values of factors influencing s.Klotho levels were analyzed. The median values are printed in bold in both the tables.

**Table 5:** Klotho group vs % Δ change at 1 year for GFR, CMIT, and albumin.

Klotho deficiency	Corresponding s.Klotho (ng/mL)	At 1 year		
		% (Δ fall GFR)	% (Δ change in CIMT)	% (Δ change in albumin)
Grade 1	> 9	< 2	< (−4.5)	< (−6.5)
Grade 2	9–6	2–13	(−4.5)–1	(−6.5)–0
Grade 3	3–5.99	13–24	1–6	0–5
Grade 4	< 3	> 24	> 6	> 5

**Table 6:** Major studies involving Klotho and CKD progression in comparison with our study.

Study	Clinical setting	Results/observations
Pavik et al. [14]	87 adults with CKD (stages 1–5) and 21 controls.	Adjusted mean Klotho decrease was 3.2 pg/mL for each 1 mL/min decrease in eGFR
Kim et al. [15]	243 adults with CKD (stages 1–5)	1. Klotho levels independently predicted the composite outcome of doubling S Cr, ESKD, or death at a median follow-up of 30 months: adjusted HR per 10 pg/mL increase, 0.96; 95% CI: 0.94–0.98; *p* < 0.001. 2. If serum Klotho was ≤ 396.3 pg/mL, 35.2% reached the composite outcome versus 15.7% if > 396.3 pg/mL (*p*=0.03). 3. Klotho levels were lower at more advanced CKD stages (*p* value for trend <0.001) and correlated positively with eGFR and negatively with FGF23 and phosphate levels.
Seiler et al. [16]	312 adults with CKD (stages 2–4)	1. Klotho levels were significantly associated with age but not with eGFR. 2. Klotho levels were not associated with the composite outcome of death or KRT initiation at a mean follow-up of 2.2 years.
Drew et al. [17]	2496 adults within the Health ABC study (mean eGFR = 73 mL/min)	Klotho levels are independently associated with a 30% kidney function decline, with each doubling of Klotho associated with 20% decreased odds of a significant decline in kidney function over 10 years.
Our study	107 patients with CKD 2–4	1. Klotho levels were significantly higher in patients with higher GFR (> 60 mL/min/1.73 m^2^) compared with advanced stages of CKD (*p* value 0.02). 2. GFR fall was significantly higher in patients with lower Klotho levels (*p* value < 0.0001). 3. Percentage fall in Klotho was strongly correlated with percentage fall in GFR (Spearman's coefficient of rank correlation (rho): 0.845 (0.778–0.892), *p* < 0.0001. 4. At a given grade of proteinuria, GFR fall was higher in patients with lower Klotho levels (observation).

## Data Availability

The data that support the findings of this study are available from the corresponding author upon reasonable request.

## Nomenclature

CKD: Chronic kidney disease
RRT: Renal replacement therapy
MBD: Mineral and bone disease
s.Klotho: Serum klotho
ABPI: Ankle brachial pressure index
CIMT: Carotid intimal medial thickness ratio
GFR: Glomerular filtration rate
SBP: Systolic blood pressure
CVD: Cardiovascular disease
ROC: Receiver operator curve
AUC: Area under curve
CRP: C reactive protein.

## Supporting Information

Supporting Table (1): Karl Pearson's correlation coefficient of s.Klotho with other parameters.
