# Supplementary material for: Chronic Kidney Disease: Decreasing Serum Klotho Levels Predict Adverse Renal and Vascular Outcomes
Source: Int J Nephrol. 2024 Nov 7;2024:2803739. doi: 10.1155/2024/2803739 (PMC11563715; doi:10.1155/2024/2803739)
Supplement: Supporting Information — Additional supporting information can be found online in the Supporting Information section. [file 2803739.f1.docx]

**Supplement Table (1): Karl Pearson’s correlation coefficient of S. Klotho with other Parameters**

| **Parameters** | **S. Klotho Initial** | | **S. Klotho Final** | | **Δ (S. Klotho)** | |
| --- | --- | --- | --- | --- | --- | --- |
|  | **r (95% CI)** | **p-value** | **r (95% CI)** | **p-value** | **r (95% CI)** | **p-value** |
| Age | -0.04 (-0.23-0.15) | 0.649 | -0.08 (-0.27-0.12) | 0.453 | -0.06 (-0.25-0.14) | 0.552 |
| eGFR Initial | 0.02 (-0.18-0.21) | 0.875 | -0.04 (-0.23-0.16) | 0.696 | 0 (-0.19-0.2) | 0.980 |
| eGFR Final | 0.09 (-0.1-0.28) | 0.347 | 0.13 (-0.06-0.32) | 0.182 | -0.22 (-0.4--0.02) | **0.028** |
| eGFR Fall | -0.32 (-0.49--0.13) | **0.002** | -0.39 (-0.55--0.2) | **<0.001** | 0.48 (0.31-0.62) | **<0.001** |
| eGFR Percent Fall | -0.47 (-0.62--0.3) | **<0.001** | -0.56 (-0.69--0.41) | **<0.001** | 0.75 (0.65-0.83) | **<0.001** |
| S. Klotho Final | 0.98 (0.96-0.98) | **<0.001** | - | - | - | - |
| Δ (S. Klotho) | -0.48 (-0.62--0.32) | **<0.001** | -0.64 (-0.74--0.51) | **<0.001** | - | - |
| HB Initial | -0.06 (-0.25-0.13) | 0.539 | -0.09 (-0.28-0.11) | 0.388 | 0.12 (-0.08-0.31) | 0.233 |
| HB Final | 0.00 (-0.19-0.2) | 0.966 | 0.00 (-0.19-0.2) | 0.987 | 0.04 (-0.16-0.23) | 0.695 |
| S. Creatinine Initial | -0.01 (-0.2-0.18) | 0.940 | 0.09 (-0.1-0.28) | 0.358 | 0.00 (-0.19-0.2) | 0.977 |
| S. Creatinine Final | 0.02 (-0.18-0.21) | 0.864 | -0.01 (-0.21-0.18) | 0.898 | 0.10 (-0.1-0.29) | 0.319 |
| Calcium Initial | -0.03 (-0.22-0.17) | 0.785 | -0.04 (-0.23-0.16) | 0.727 | 0.02 (-0.18-0.22) | 0.842 |
| Calcium Final | -0.03 (-0.23-0.16) | 0.739 | -0.02 (-0.22-0.17) | 0.813 | 0.00 (-0.2 - 0.19) | 0.966 |
| PO Initial | 0.10 (-0.1-0.29) | 0.336 | 0.14 (-0.06-0.33) | 0.169 | -0.09 (-0.28-0.11) | 0.398 |
| PO Final | 0.03 (-0.17-0.22) | 0.800 | 0.04 (-0.16-0.24) | 0.680 | -0.09 (-0.28-0.11) | 0.390 |
| Product Ratio Ca*PO | 0.13 (-0.07-0.32) | 0.207 | 0.16 (-0.04-0.35) | 0.114 | -0.08 (-0.28-0.12) | 0.433 |
| Albumin Initial | 0.11 (-0.09-0.3) | 0.279 | 0.06 (-0.14-0.25) | 0.580 | 0.00 (-0.2-0.2) | 0.985 |
| Albumin Final | 0.36 (0.17-0.52) | **<0.001** | 0.39 (0.21-0.55) | **<0.001** | -0.4 (-0.56--0.22) | **<0.001** |
| Cholesterol | 0.13 (-0.2-0.43) | 0.449 | 0.13 (-0.21-0.44) | 0.459 | -0.11 (-0.42-0.23) | 0.525 |
| LDL | 0.07 (-0.26-0.39) | 0.681 | 0.04 (-0.29-0.36) | 0.815 | 0.11 (-0.23-0.42) | 0.526 |
| HDL | 0.00 (-0.33-0.32) | 0.988 | -0.01 (-0.34-0.32) | 0.956 | -0.04 (-0.36-0.3) | 0.831 |
| Triglyceride | -0.04 (-0.35-0.29) | 0.824 | -0.12 (-0.43-0.21) | 0.482 | 0.17 (-0.16-0.47) | 0.309 |
| FBS | -0.23 (-0.64-0.29) | 0.383 | -0.25 (-0.66-0.28) | 0.346 | 0.33 (-0.2-0.71) | 0.219 |
| HbA1c | 0.04 (-0.25-0.31) | 0.808 | 0.03 (-0.26-0.31) | 0.855 | -0.04 (-0.32-0.24) | 0.776 |
| CRP Initial | -0.24 (-0.42 - -0.05) | **0.016** | -0.23 (-0.41 - -0.03) | **0.025** | 0.35 (0.16-0.51) | **0.001** |
| CRP Final | -0.21 (-0.39--0.01) | **0.044** | -0.22 (-0.40 - -0.02) | **0.034** | 0.34 (0.15-0.51) | **0.001** |
| Urine Protein | -0.12 (-0.33-0.1) | 0.294 | -0.11 (-0.33-0.11) | 0.323 | 0.06 (-0.16-0.28) | 0.603 |
| PTH | -0.02 (-0.22 - 0.19) | 0.860 | 0.09 (-0.12-0.3) | 0.403 | -0.18 (-0.37-0.03) | 0.100 |
| Ferritin Initial | 0.05 (-0.15-0.24) | 0.615 | 0.02 (-0.18-0.21) | 0.861 | 0.06 (-0.14-0.25) | 0.549 |
| Ferritin Final | -0.16 (-0.35-0.04) | 0.112 | -0.18 (-0.37-0.01) | 0.066 | 0.16 (-0.04-0.35) | 0.107 |
| Saturation % Initial | 0.03 (-0.23-0.28) | 0.832 | 0.04 (-0.23-0.3) | 0.793 | -0.11 (-0.36-0.16) | 0.418 |
| Saturation % Final | -0.02 (-0.26-0.23) | 0.901 | -0.02 (-0.26-0.23) | 0.903 | -0.05 (-0.29-0.2) | 0.704 |
| SBP Initial | -0.44 (-0.58 - -0.27) | **<0.001** | -0.46 (-0.6 - -0.29) | **<0.001** | 0.38 (0.2-0.54) | **<0.001** |
| SBP Final | -0.21 (-0.39 - -0.02) | **0.031** | -0.19 (-0.38-0) | 0.052 | 0.26 (0.06-0.43) | **0.010** |
| ABPI Initial | 0.44 (0.27-0.58) | **<0.001** | 0.45 (0.28-0.6) | **<0.001** | -0.5 (-0.63 - -0.33) | **<0.001** |
| ABPI Final | 0.33 (0.15-0.49) | **0.001** | 0.4 (0.22-0.55) | **<0.001** | -0.47 (-0.61 - -0.3) | **<0.001** |
| CIMT Initial | -0.5 (-0.63 - -0.34) | **<0.001** | -0.53 (-0.66 - -0.38) | **<0.001** | 0.53 (0.38-0.66) | **<0.001** |
| CIMT Final | -0.53 (-0.66 - -0.38) | **<0.001** | -0.57 (-0.69 - -0.43) | **<0.001** | 0.58 (0.43-0.7) | **<0.001** |
| Uric Acid | 0.06 (-0.14-0.26) | 0.536 | 0.13 (-0.08-0.32) | 0.236 | -0.06 (-0.26-0.15) | 0.606 |
| **Note that; r = Pearson Correlation coefficient; CI = Confidence Interval** | | | | | | |

Karl – Pearson’s correlation coefficient used to measure amount of correlation between S.klotho and other quantitative parameters. P-value < 0.05 considered to be significant correlation at 5% level of significance.
